# Supplementary material for: Elevated plasma progranulin levels in the acute phase are correlated with recovery of left ventricular function in the chronic phase in patients with acute myocardial infarction
Source: PLoS One. 2024 Nov 8;19(11):e0313014. doi: 10.1371/journal.pone.0313014 (PMC11548716; doi:10.1371/journal.pone.0313014)
Supplement: S1 File — (DOCX) [file pone.0313014.s002.docx]

2 April 2019 submission

**Application form for ethical committee of medical research**

Director of Gifu Municipal Hospital

Applicant

　　　　　　　　　　　　　　　　　 Affiliation: Heart center

Job title: Head of heart center

Name of research representative:

Shinya Minatoguchi

**Protocol （progranulin study）**

**Subjects:** Cardiac patients with complaints of anterior chest who underwent coronary angiography.

**Control group and acute myocardial infarction（AMI）group (n-20 each):**

Out of cardiac patients who underwent coronary angiography, patients without significant coronary angiography are assigned to control group, and patients with total coronary occlusion and electrocardiography abnormality and biochemical blood test abnormaliy are assigned to AMI group.

**Method:** In AMI group, 1 mL of blood is taken from the vein and plasma progranulin level is measured by ELIZA on day 1 and day 7 of admission. Cardiac function is measured within 7 days after admission and 6 months after AMI. In control group, plasma progranulin level is measured by ELIZA and cardiac function is measured by echocardiography during admission.

**Assessment:** The behavior of plasma progranulin level is compared between the control and AMI groups. In AMI group, relationship between changes in plasma progranulin level and changes in cardiac function in the chronic phase (6 months) is assessed.
